# An unusual case report of porokeratosis ptychotropica on the buttocks

**DOI:** 10.1097/MD.0000000000032074

**Published:** 2022-11-25

**Authors:** Yu Xiao, Shanshan Peng, Tianyi Mao, Xiangjun Li, Wenzheng Ye, Muping Fang

**Affiliations:** a The Central Hospital of Xiaogan, Xiaogan Hospital Affiliated to Wuhan University of Science and Technology, Hubei, China; b The Central Hospital of Xiaogan, the Central Hospital of Xiaogan, Jinzhou Medical University, Hubei, China.

**Keywords:** diagnosis, histology, porokeratosis

## Abstract

Porokeratosis ptychotropica represents an unusual form of porokeratosis characterized by symmetrical dyskeratotic skin lesions on the gluteal clefts. Herein, we report a case of porokeratosis ptychotropica.

**Patient concerns:**

A 33-year-old man, who complained of itching papules and plaques in the gluteal cleft and the buttocks for the last 7 years. Clinical examination showed a large well-defined reddish brown verrucous plaque located on both buttocks along with satellite papules on the inner thigh. Dermoscopy and histopathological findings were consistent with porokeratosis.

**Diagnosis:**

He was diagnosed with porokeratosis ptychotropica.

**Outcomes:**

No significant improvement was observed following treatment with oral acitretin and a topical retinoid.

**Lessons:**

The case report highlights the need for awareness amongst dermatologists for porokeratosis ptychotropica as a differential diagnosis for pruritic papules in the gluteal fold.

## 1. Introduction

Porokeratosis is a spectrum of clinical disorders characterized by the histological features of a cornoid lamella. Several variants have been described, including linear porokeratosis, classical porokeratosis of Mibeli, disseminated superficial actinic porokeratosis, and prokeratosis plantaris and palmaris.^[1]^ Interestingly, another rare variant, porokeratosis ptychotropica, localized to the gluteal cleft and the buttocks, was first reported by the Lucker et al in 1995.^[2]^ Herein, we present the case report of a patient with porokeratosis ptychotropica, an unusual diagnosis, and describe the findings of clinical routine, dermoscopy and histology.

## 2. Case report

A 33-year-old Chinese male presented with a 7-year history of itching brown papules and plaques on the gluteal cleft and the buttocks. The lesion began as a small brown scaly papule and increasingly coalesced to form larger plaques. There was no discernable history or underlying cutaneous diseases. He did not respond to treatment with topical glucocorticoid and antihistamine drugs. Physical examination revealed a large well-defined reddish brown verrucous plaque located on both buttocks, and satellite papules were found at the inner thigh (Figs. 1 and 2). Demoscopy showed a sharply demarcated annular lesion with a thick, peripheral light brown rim, limiting an erythematous non-atrophic center with regular dotted vessels (Fig. 3). Skin biopsy showed psoriasiform hyperplasia, multiple cornoid lamellae, and an absence of amyloid deposition (Fig. 4). A diagnosis of porokeratosis ptychotropica was considered based on the clinical findings and typical histological features. Treatment with the administration of oral acitretin and application of topical retinoid creams daily was prescribed, but the rash showed no significant improvement during 4-month follow up. We obtained written informed consent from the patient for publication of their data and accompanying images.

**Figure 1.**
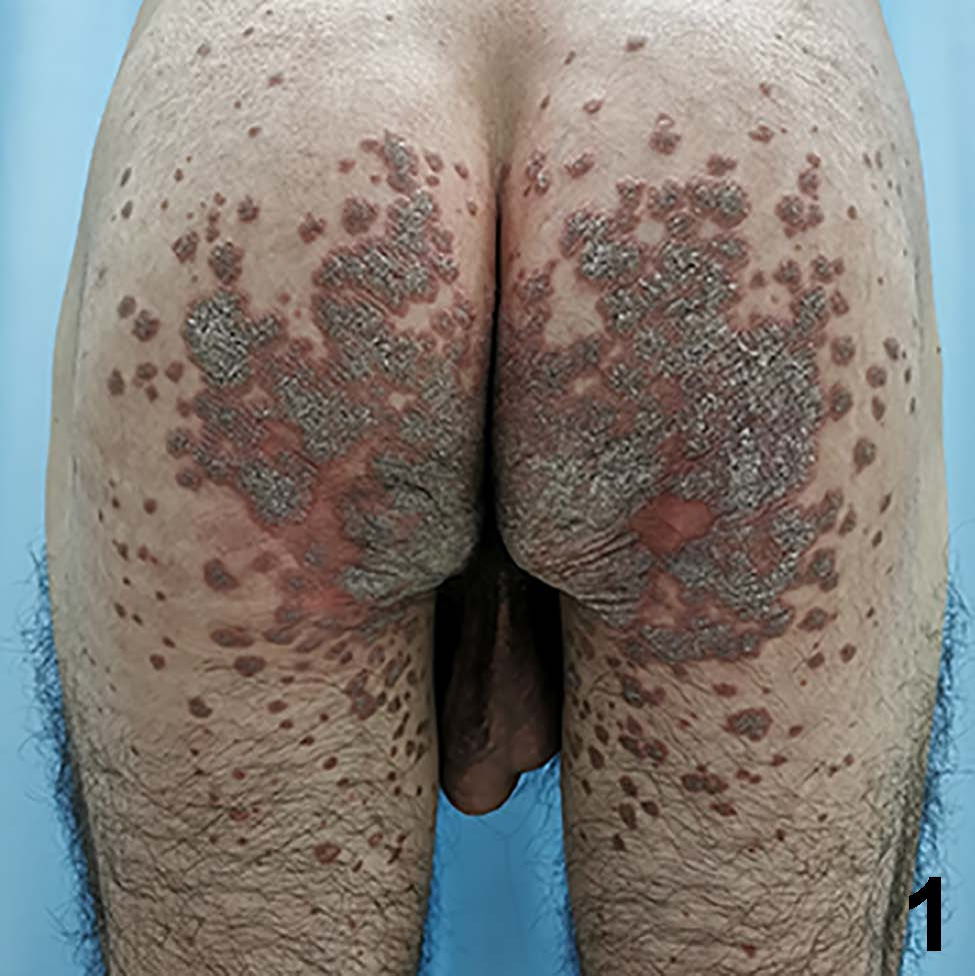
2. A reddish-brown, well-demarcated, hypertrophic and invasive verrucous plaque was presented bilaterally and symmetrically on the buttocks.

**Figure 2.**
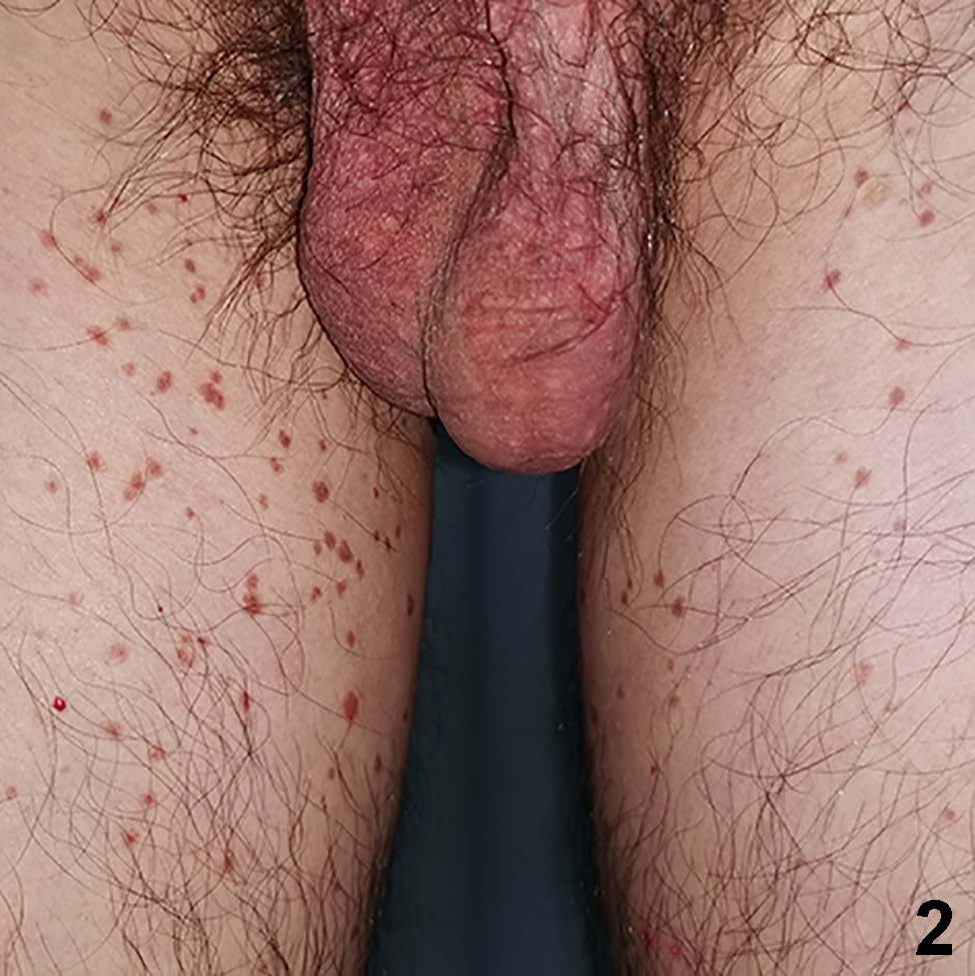
Numerous well-defined reddish brown discrete papules were found symmetrically at the inner thigh.

**Figure 3.**
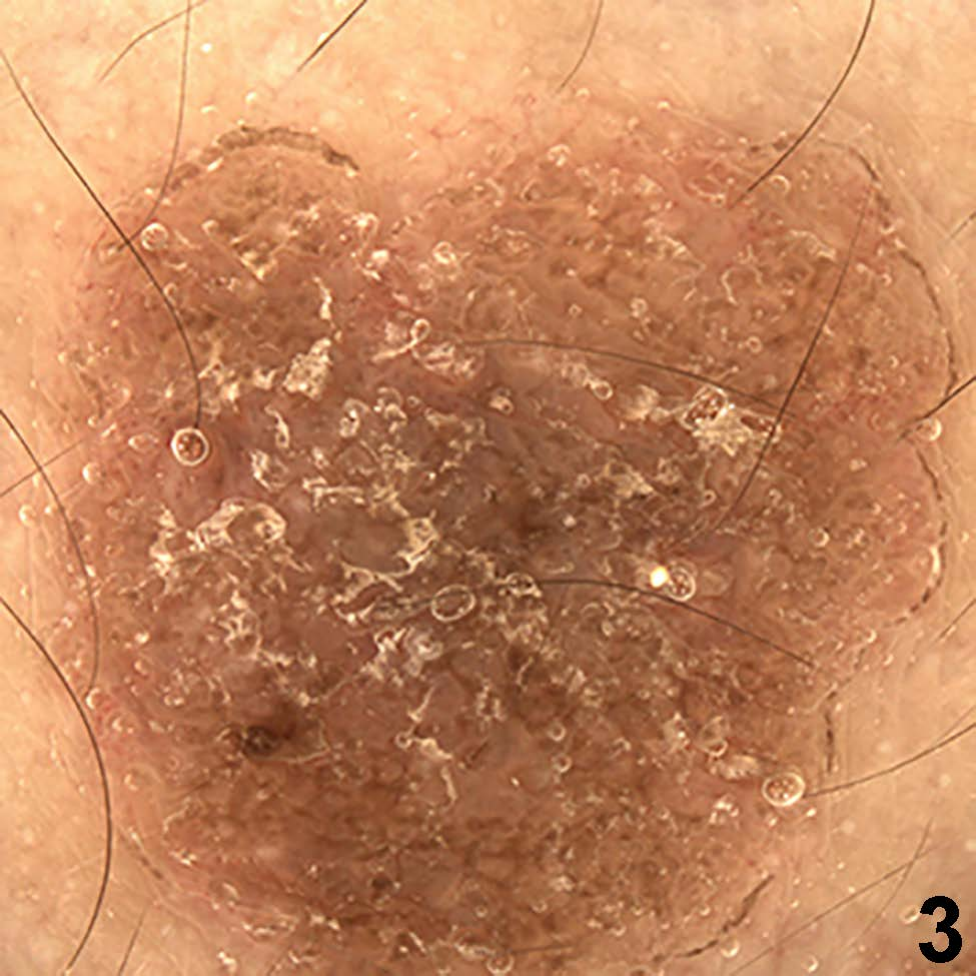
A sharply demarcated annular lesion with a thick, peripheral light brown rim, limiting an erythematous non-atrophic center with regular dotted vessels.

**Figure 4.**
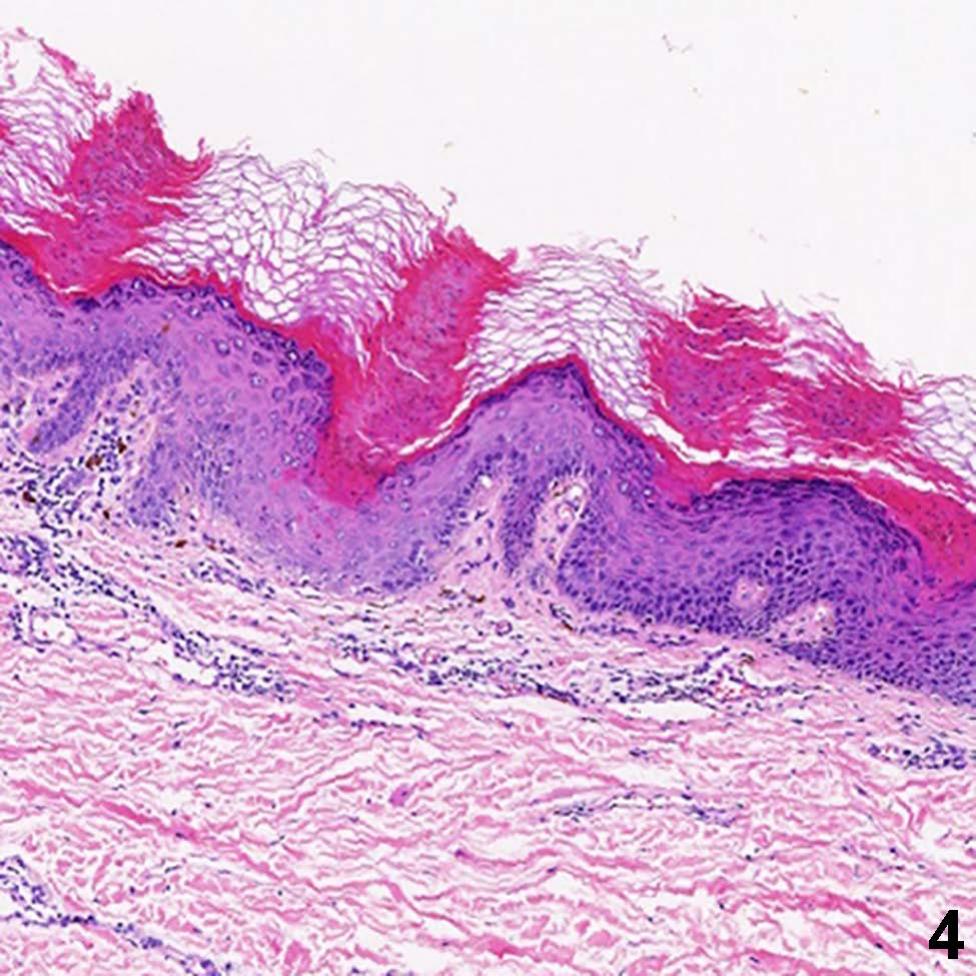
Epidermal hyperkeratosis and parakeratosis with cornoid lamellae in the stratum corneum (HE × 100). HE = human immunodeficiency virus.

## 3. Discussion

Porokeratosis ptychotropica was previously referred to as verrucous porokeratosis or genitogluteal porokeratosis is rare.^[1,3,4]^ Porokeratosis ptychotropica the preferred term, is localized to the gluteal folds, buttocks and genitalia with a butterfly morphology.^[1]^ It increasingly progresses through satellite lesions that develop and coalesce, accompanied by mild-to-severe itching. Moreover, this disease is more likely to occur in males, as in the present case.

To date, several scholars have suggested that numerous factors are associated with porokeratosis ptychotropica, including repeated trauma, organ transplantation, HIV infection and other diseases related to immunosuppression, but its pathogenesis remains unclear.^[1,5]^ Interestingly, none of these factors were implicated in our case. The prevalence of malignant transformation of the porokeratotic rash is 7.5%, especially the linear and large-sized lesions. Previously, a case of a 6-year-porokeratosis ptychotropica transforming into invasive squamous cell carcinoma has been reported.^[6]^ Periodic follow up plays a vital role in the early diagnosis of potential transformation, especially to malignant basal cell carcinoma, squamous cell carcinomas or malignant melanoma.^[7]^

Dermoscopic findings revealed a sharply demarcated annular lesion with a thick, peripheral light brown rim, limiting an erythematous non-atrophic center with regular dotted vessels, which were vital clues suggesting a subtype of porokeratosis.^[8]^ but not a specific manifestations of porokeratosis ptychotropica.

Among histological features, the differences between porokeratosis ptychotropica and other variants are in the number and distribution of the cornoid lamellae.^[9]^ Cornoid lamellae are present throughout the lesion in porokeratosis ptychotropica, while in the other variants, these are localized to the periphery. Multiple of cornoid lamellae were present in our patient.

Differential diagnosis includes psoriasis, tuberculosis verrucosa cutis, and lichen planus verrucose. Numerous conventional treatments, including acitretin, corticosteroids, 5-fluorouracil, cryotherapy, and laser therapy are available but their efficacy is limited for porokeratosis ptychotropica.^[10,11]^ Surgical excision may be a good option for early-stage lesions.^[10,11]^

In summary, dermoscopic findings are helpful, but the clinical characteristics and histological examinations have significant roles in the definite diagnosis of porokeratosis ptychotropica. This case report is expected to raise awareness of porokeratosis ptychotropica, an identified diagnosis variant of porokeratosis. We also hope to improve diagnostic accuracy and prevent the long-term complications related to the disease.

## Acknowledgments

The patients in this case have given written informed consent to publication of her case details.

## Author contributions

**Conceptualization:** Yu Xiao, Xiangjun Li, Wenzheng Ye, Muping Fang.

**Data curation:** Yu Xiao, Shanshan Peng, Tianyi Mao, Xiangjun Li, Wenzheng Ye, Muping Fang.

**Formal analysis:** Yu Xiao, Wenzheng Ye, Muping Fang.

**Funding acquisition:** Yu Xiao.

**Investigation:** Yu Xiao.

**Methodology:** Yu Xiao.

**Resources:** Yu Xiao.

**Software:** Yu Xiao, Muping Fang.

**Supervision:** Shanshan Peng, Xiangjun Li, Wenzheng Ye, Muping Fang.

**Validation:** Yu Xiao, Muping Fang.

**Visualization:** Yu Xiao, Muping Fang.

**Writing – original draft:** Yu Xiao, Shanshan Peng, Tianyi Mao, Wenzheng Ye, Muping Fang.

**Writing – review & editing:** Yu Xiao, Wenzheng Ye, Muping Fang.
